# Epidermal NAD^+^ deficiency induces IL-36–mediated skin inflammation and acanthosis

**DOI:** 10.1172/jci.insight.189177

**Published:** 2026-02-10

**Authors:** Taiki Seki, Jun-Dal Kim, Yasuhito Yahara, Hitoshi Uchida, Keisuke Yaku, Mariam Karim, Teruhiko Makino, Tadamichi Shimizu, Takashi Nakagawa

**Affiliations:** 1 Department of Molecular and Medical Pharmacology, Faculty of Medicine,; 2 Department of Dermatology, Faculty of Medicine, and; 3 Division of Complex Biosystem Research, Department of Research and Development, Institute of Natural Medicine, University of Toyama, Toyama, Japan.; 4 AMED-CREST, Japan Agency for Medical Research and Development, Chiyoda-ku, Tokyo, Japan.; 5 Department of Orthopedic Surgery, Faculty of Medicine, University of Toyama, Toyama, Japan.; 6 Department of Oral Chrono-Physiology, Nagasaki University Graduate School of Biomedical Sciences, Nagasaki, Japan.; 7 Research Center for Pre-Disease Science, University of Toyama, Toyama, Japan.

**Keywords:** Dermatology, Metabolism, Cytokines, DNA repair

## Abstract

Nicotinamide adenine dinucleotide (NAD^+^) is essential for cellular metabolism, DNA repair, and stress responses. NAD^+^ is synthesized from nicotinamide, nicotinic acid (collectively termed niacin), and tryptophan. In humans, deficiencies in these nutrients result in pellagra, marked by dermatitis, diarrhea, and dementia. The dermatitis associated with pellagra typically manifests as photodermatosis in sun-exposed areas. This study examined the effects of NAD^+^ deficiency on skin homeostasis using epidermis-specific Nampt–conditional KO (Nampt-cKO) mice. These mice displayed substantial NAD^+^ depletion, reduced poly(ADP-ribose) polymerase (PARP) activity, and increased DNA damage. Consequently, Nampt-cKO mice developed spontaneous skin inflammation and epidermal hyperplasia. RNA-seq and IHC analyses demonstrated increased IL-36 cytokine expression, suggesting that DNA repair–related genomic stress triggers keratinocyte-driven IL-36 production, which promotes inflammation. Furthermore, reduced COL17A1 expression and elevated thymic stromal lymphopoietin (TSLP) levels were observed. NAD^+^ repletion by transdermal supplementation of nicotinamide mononucleotide (NMN) suppressed the rise of IL-36 levels and skin inflammation. These findings underscore the importance of Nampt-mediated NAD^+^ metabolism for epidermal stability and indicate that NAD^+^ depletion may contribute to IL-36–mediated skin inflammation, offering insights for therapeutic strategies in inflammatory skin disorders.

## Abstract 2

Epidermal loss of Nampt causes a marked reduction in NAD^+ ^levels, PARP inactivation, and increased DNA damage, leading to spontaneous skin inflammation and keratinocyte hyperproliferation.

## Graphical abstract

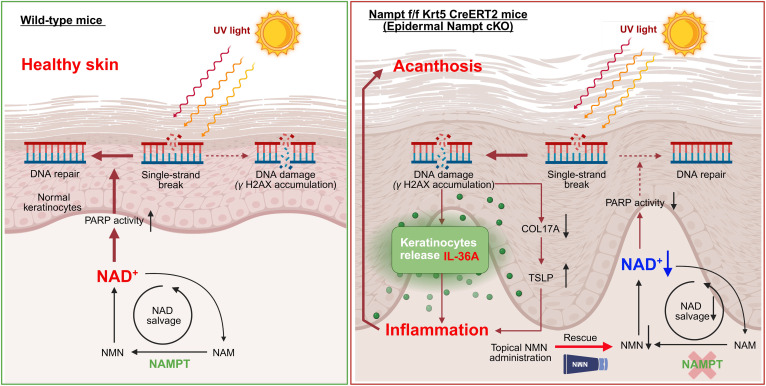

## Introduction

Nicotinamide adenine dinucleotide (NAD^+^) is a vital coenzyme that supports numerous cellular functions, including energy metabolism, DNA repair, and stress responses (1, 2). As a key factor in redox reactions, NAD^+^ facilitates electron transfer in metabolic pathways like glycolysis, the tricarboxylic acid cycle, and oxidative phosphorylation, which contribute to adenosine triphosphate (ATP) production (1). Beyond metabolism, NAD^+^ also acts as a substrate for enzymes such as sirtuins, poly(ADP-ribose) polymerases (PARPs), CD38, and SARM1, influencing DNA repair, genomic stability, gene expression, cell survival, calcium mobilization, and axon degeneration (1, 2). NAD^+^ is synthesized from nicotinamide (NAM), nicotinic acid, and tryptophan via the salvage, Preiss-Handler, and de novo pathways, respectively. NAM riboside (NR) and NAM mononucleotide (NMN) are alternative dietary precursors for the salvage pathway (3). NAM phosphoribosyltransferase (Nampt) is a rate-limiting enzyme in the salvage pathway, converting NAM to NMN (4). Therefore, Nampt is considered critical for maintaining intracellular NAD^+^ levels. Notably, NAD^+^ levels have been found to decline with age across various tissues, leading to reductions in energy metabolism, mitochondrial function, and cellular homeostasis (5, 6). Furthermore, administration of NAD^+^ precursors, such as NR and NMN, increases NAD^+^ levels and exhibits beneficial effects against aging and aging-related disorders (7, 8).

The skin, particularly the epidermis, is continually exposed to environmental stressors like ultraviolet (UV) radiation, pathogens, and chemicals. To sustain its barrier function and homeostasis, the epidermis depends on rapid cell turnover and robust stress response mechanisms (9). In this context, NAD^+^ is crucial for protecting epidermal cells from UV-induced damage by maintaining energy levels and facilitating DNA repair. During UV exposure, NAD^+^ is quickly depleted as PARP enzymes are activated for DNA repair, but Nampt replenishes NAD^+^ to aid cell survival (10). Pellagra, a disease resulting from deficiency of niacin (alternatively called vitamin B3) or disruptions in its metabolism, is now rare due to better nutrition but still occurs in cases of severe malnutrition and alcohol dependency (11). Characterized by the “3 Ds” — dermatitis, diarrhea, and dementia — pellagrous dermatitis is notable for its photosensitivity. Since pellagra can also arise from tryptophan deficiency, another NAD^+^ source, it is thought that reduced NAD^+^ levels may contribute to its pathology. However, the exact mechanism by which NAD^+^ deficiency leads to pellagra remains unclear.

Unlike pellagra, chronic inflammatory skin conditions such as psoriasis and atopic dermatitis are characterized by elevated NAD^+^ levels and increased Nampt activity (12–14). This NAD^+^ excess, driven by Nampt overexpression, promotes the production of proinflammatory cytokines and keratinocyte hyperproliferation, intensifying inflammation and immune dysregulation (15). In psoriasis, Nampt is upregulated in both lesional skin and immune cells, contributing to the amplification of inflammation (16). Similarly, Nampt overexpression in atopic dermatitis connects immunometabolic dysfunction with persistent inflammation (17). While NAD^+^ deficiency can trigger inflammation and structural deterioration, as seen in pellagra, excessive Nampt activity may fuel pathological inflammation in other inflammatory skin disorders. These findings imply the dual role of NAD^+^ metabolism in skin health.

To examine the physiological and pathophysiological role of NAD^+^ metabolism in the skin, we generated tamoxifen-inducible, epidermis-specific Nampt–conditional KO (Nampt-cKO) mice. Loss of Nampt in the epidermis led to a marked reduction in NAD^+^ levels, PARP inactivation, and increased DNA damage. These damages in Nampt-cKO mice induced IL-36 in the epidermis, developing spontaneous skin inflammation and keratinocyte hyperproliferation. Furthermore, percutaneous administration of NMN in Nampt-cKO mice effectively restored NAD^+^ levels and suppressed IL-36 production, thereby attenuating the subsequent skin inflammation. These findings highlight the crucial role of NAD^+^ metabolism in skin health and give a clue for unveiling the pathophysiology of photosensitive dermatitis, such as pellagra.

## Results

### Epidermal NAD^+^ depletion induces spontaneous skin inflammation.

**Figure 1:**
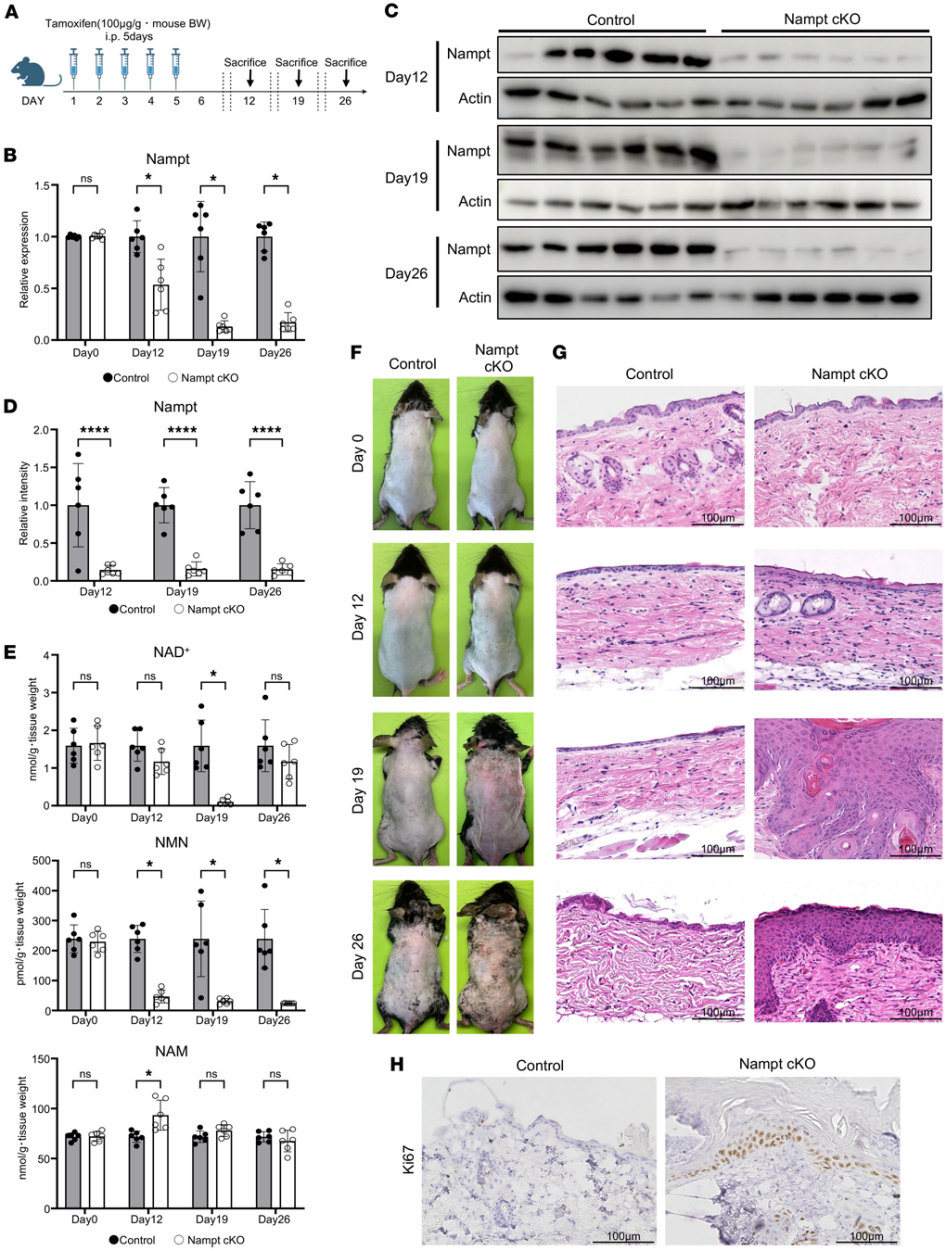
Spontaneous skin inflammation due to NAD^+^ depletion in Nampt-cKO mice. (**A**) Experimental time line showing tamoxifen administration and sample collection for control and Nampt-cKO mice. (**B**) Nampt mRNA levels in the epidermis of control and Nampt-cKO mice on Days 0, 12, 19, and 26, as determined by qPCR. Data are shown as mean ± SEM. Student’s *t* test. *n* = 6. (**C**) Western blot analysis of Nampt in epidermal lysates from control and Nampt-cKO mice at Days 12, 19, and 26 with pan-actin as a loading control. (**D**) Semiquantitative densitometric analysis of Nampt Western blots normalized to pan-actin using ImageJ. Data are shown as mean ± SEM. Student’s *t* test., *n* = 6. (**E**) NAD^+^, NMN, and NAM levels in control and Nampt-cKO epidermis on Days 0, 12, 19, and 26, as measured by LC/MS. Data are shown as mean ± SEM. Student’s *t* test. *n* = 6. (**F**) Representative clinical photographs of control and Nampt-cKO skin at Days 0, 12, 19, and 26. (**G**) Representative H&E-stained sections of control and Nampt-cKO skin at Days 0, 12, 19, and 26. Scale bars: 100 μm. (**H**) Ki67 immunohistochemistry in control and Nampt-cKO epidermis on Day 19. Scale bars: 100 μm. *P < 0.05, ****P < 0.0001.

To investigate the role of NAD^+^ metabolism in epidermal homeostasis, we created tamoxifen-induced, epidermis-specific Nampt-cKO mice by crossing Nampt flox mice with Krt5-CreERT2 mice. Tamoxifen administration effectively reduced Nampt mRNA levels in the epidermis of Nampt-cKO mice from Day 12, with a marked reduction by Day 19 compared with that of control mice (Figure 1, A and B). Consistently, protein levels of Nampt in epidermis were significantly reduced in Nampt-cKO mice at Day 19 (Figure 1, C and D). This decrease in Nampt led to significantly reduced NAD^+^ and NMN levels in the skin by Day 12, with further depletion by Day 19 (Figure 1E). In contrast, NAM levels were slightly increased in Nampt-cKO mice at Day 12 (Figure 1E). Starting around Day 19 after tamoxifen treatment, Nampt-cKO mice developed erythema, skin thickening, and crust formation (Figure 1F). Histological analysis on Day 19 showed epidermal hyperplasia, necrosis, and notable infiltration of inflammatory cells in the dermis (Figure 1G). This epidermal thickening was associated with increased proliferation in the epidermis, as shown by a rise in Ki67^+^ cells (Figure 1H). By Day 26, the inflammation began to resolve, although the epidermal thickening persisted (Figure 1, F and G). Therefore, we examined NAD^+^ levels at Day 26 and found that NAD^+^ levels were recovered in Nampt-cKO mice (Figure 1E). We also revealed that Nampt expression and NMN levels were still low in Nampt-cKO mice at Day 26 (Figure 1, B–E), suggesting that other synthetic routes, such as de novo and Preiss-Handler pathways, increased NAD^+^ levels in a compensatory manner.

### IL-36–mediated inflammation without T cell polarization in Nampt-cKO mice.

**Figure 2:**
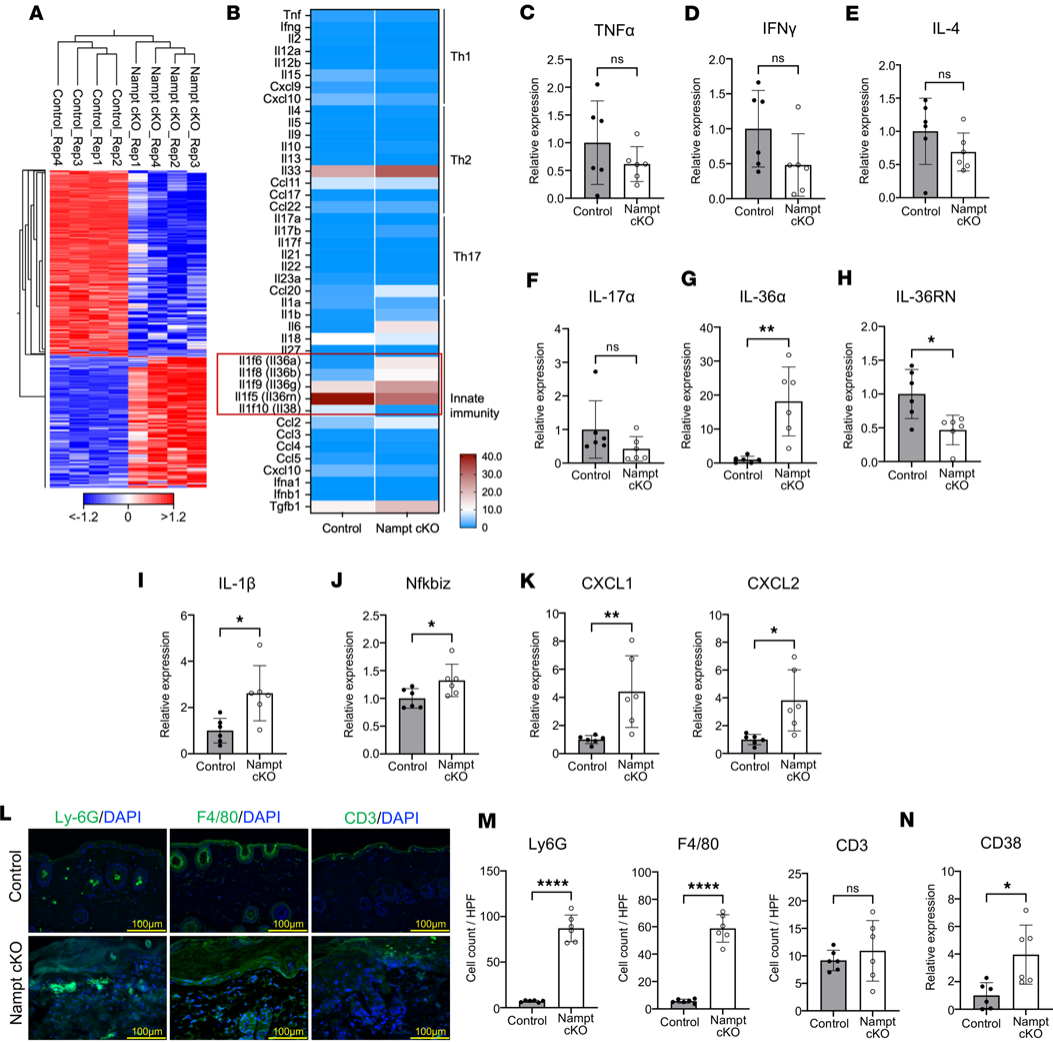
IL-36–driven inflammation without T cell polarization in Nampt-cKO mice. (**A**) RNA-seq heatmap for gene expression in control and Nampt-cKO skin on Day 19. *n* = 4. (**B**) Heatmap of individual inflammatory cytokine expression levels from RNA-seq data. (**C**–**K**) Gene expression levels of TNF-α (**C**), IFN-γ (**D**), IL-4 (**E**), IL-17α (**F**), IL-36α (**G**), IL-36RN (**H**), IL-1β(**I**), Nfkbiz (**J**), and CXCL1 and CXCL2 (**K**) in control and Nampt-cKO skin on Day 19, as determined by qPCR. Data are shown as mean ± SEM. Student’s *t* test. *n* = 6. (**L**) Representative immunofluorescence images for Ly-6G (neutrophils), F4/80 (macrophages), and CD3 (T cells) in control and Nampt-cKO skin on Day 19. Nuclei were counterstained with DAPI. Scale bars: 100 μm. (**M**) Quantification of Ly-6G^+^, F4/80^+^, and CD3^+^ cells using ImageJ. Data are shown as mean ± SEM. Student’s *t* test. *n* = 6. (**N**) Gene expression level of CD38 in control and Nampt-cKO skin on Day 19, as determined by qPCR. Data are shown as mean ± SEM. 2-tailed Student’s *t* test. *n* = 6. *P < 0.05, **P < 0.01, ****P < 0.0001.

To examine the inflammatory response resulting from Nampt deficiency, we conducted an RNA-seq analysis of skin tissues from Nampt-cKO mice on Day 19 (Figure 2A). The results showed no apparent change of genes related to Th1, Th2, or Th17 differentiation, while genes related to IL-36 signaling pathway were significantly altered (Figure 2B). qPCR analysis further confirmed that cytokine levels of TNF-α, IFN-γ, IL-4, and IL-17A were not significantly different between Nampt-cKO and control mice (Figure 2, C–F). However, a notable increase in IL-36 family cytokines (IL-36α) was observed in Nampt-cKO mice (Figure 2G). Correspondingly, there was a downregulation of IL-36RN, which acts as an IL-36 receptor antagonist (Figure 2H). IL-36, a proinflammatory cytokine of the IL-1 family, is expressed in epithelial cells, including keratinocytes, and abnormal activation of IL-36 signaling pathway is associated with various inflammatory skin diseases (18). Generalized pustular psoriasis (GPP) is specifically linked to IL-36RN mutations, which activate IL-36 signaling (19). Several transcription factors, including NF-κB, AP-1, and T-bet, have been implicated in the induction of IL-36A, and these pathways are activated downstream of various proinflammatory cytokines, such as IL-1β, TNF-α, IFN-γ, IL-18, and IL-17A (20). To identify the upstream signals driving IL-36A expression in Nampt-cKO mice, we examined the expression of cytokines known to induce IL-36A and found a marked upregulation of IL-1β (Figure 2I). We also observed a pronounced increase in IκBζ (NFKBIZ) (Figure 2J), a transcriptional coactivator that amplifies NF-κB signaling (21). Importantly, IκBζ functions not only upstream to promote IL-36A induction but also downstream of IL-36A (22), where it is required for the expression of the chemokines CXCL1 and CXCL2, both of which were markedly elevated in Nampt-cKO skin (Figure 2K). Patients with GPP show a marked infiltration of innate immune cells, such as neutrophils and macrophages, rather than adaptive immune cells (19). Furthermore, CXCL1 and CXCL2 are potent chemokines that strongly recruit neutrophils. Therefore, we investigated the infiltrated immune cells in Nampt-cKO mice. Immunostaining with Ly-6G (neutrophils) and F4/80 (macrophages) antibodies confirmed significant neutrophil and macrophage infiltration in the skin of Nampt-cKO mice (Figure 2, L and M). However, CD3 (T cell) staining showed no significant difference between Nampt-cKO and control mice (Figure 2, L and M). We also found that gene expression of CD38 was upregulated in the epidermis of Nampt-cKO mice (Figure 2N). CD38 has been shown as a marker of M1 macrophage, a type of proinflammatory macrophage (23). As CD38 hydrolyzes NAD^+^ to NAM, it may also be responsible for reducing NAD^+^ levels during inflammation. These findings indicate that skin inflammation in Nampt-cKO mice is driven by IL-36 signaling activation, leading to an innate immune response.

### Accumulation of DNA damage due to reduced PARP activity in Nampt-cKO mice.

**Figure 3:**
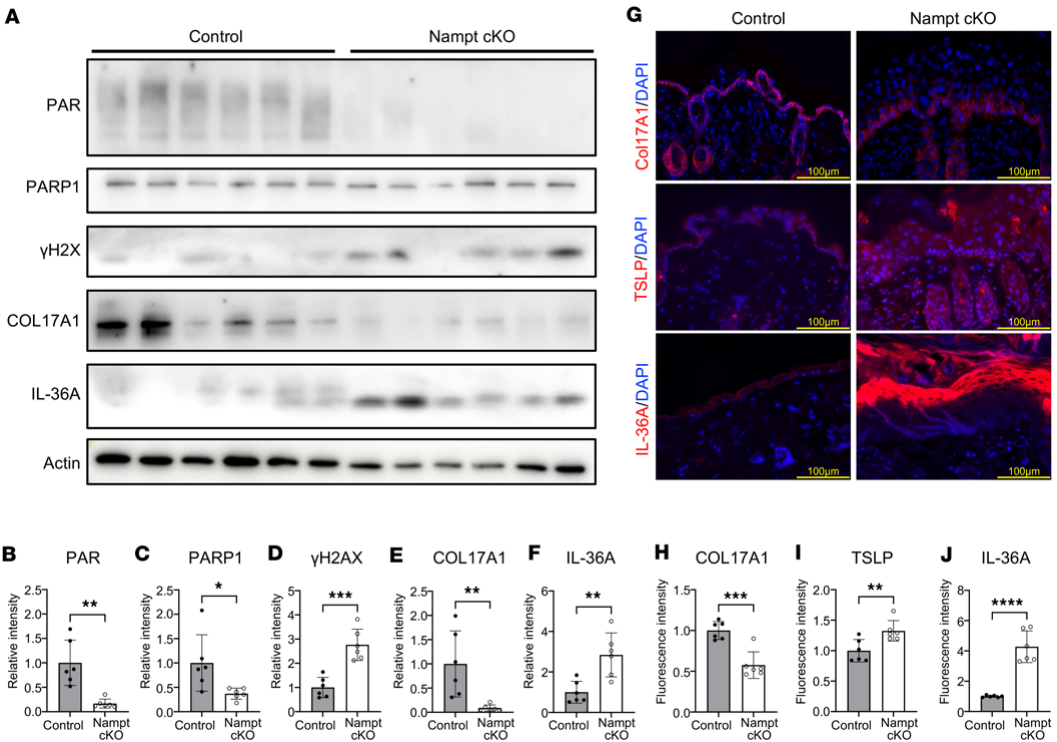
DNA Damage accumulation from reduced PARP activity in Nampt-cKO mice. (**A**) Western blot analysis for PAR, PARP1, γH2AX, COL17A1, and IL-36A in control and Nampt-cKO skin on Day 19, with pan-actin as a loading control. (**B**–**F**) Semiquantitative densitometric analysis of Western blots for PAR (**B**), PARP1 (**C**), γH2AX (**D**), COL17A1 (**E**), and IL-36A (**F**), normalized to pan-actin using ImageJ. Data are shown as mean ± SEM. Student’s *t* test. *n* = 6. (**G**) Representative immunofluorescence images of TSLP, COL17A1, and IL-36A in control and Nampt-cKO skin on Day 19. Scale bars: 100 μm. (**H**–**J**) Quantification of COL17A1 (**H**), TSLP (**I**), and IL-36A (**J**) in immunofluorescence images using ImageJ. Data are shown as mean ± SEM. 2-tailed Student’s *t* test. *n*= 6. *P < 0.05, **P < 0.01, ***P < 0.001, ****P < 0.0001.

We explored how NAD^+^ deficiency led to IL-36 expression in the epidermis. Considering the role of NAD^+^ in DNA repair (24), we assessed PARP activity and DNA damage markers in Nampt-cKO mice. Western blot analysis revealed a significant reduction in PARP1 and its product, poly-ADP ribose (PAR), in the skin of Nampt-cKO mice (Figure 3, A–C), indicating impaired PARP function due to NAD^+^ depletion. Moreover, the DNA damage marker γH2AX was notably elevated in Nampt-cKO mice, as shown by Western blot (Figures 3, A and D). These findings indicate that NAD^+^ deficiency causes DNA repair defects and genomic stress accumulation in keratinocytes. A previous study showed that genomic stress leads to the proteolysis of collagen, type XVII, α 1 (COL17A1), impairing epidermal stem cell function (25, 26). Therefore, we examined COL17A1 expression in Nampt-cKO mice. Western blot analysis revealed reduced COL17A1 levels in these mice (Figure 3, A and E). Additionally, IL-36A expression was increased in the epidermis of Nampt-cKO mice (Figure 3, A and F). Previous research has shown that thymic stromal lymphopoietin (TSLP), another epithelial cell–derived cytokine, is induced by COL17A1 dysfunction (27). Immunostaining analysis confirmed that COL17A1 expression was decreased in Nampt-cKO mice (Figure 3, G and H). In contrast, the levels of TSLP and IL-36A were significantly elevated in these mice (Figure 3, G, I, and J). These results suggest that NAD^+^ depletion triggers IL-36A and TSLP expression through epithelial dysfunction and COL17A1 proteolysis.

### Photoactivation enhances epidermal inflammation in Nampt-cKO mice.

**Figure 4:**
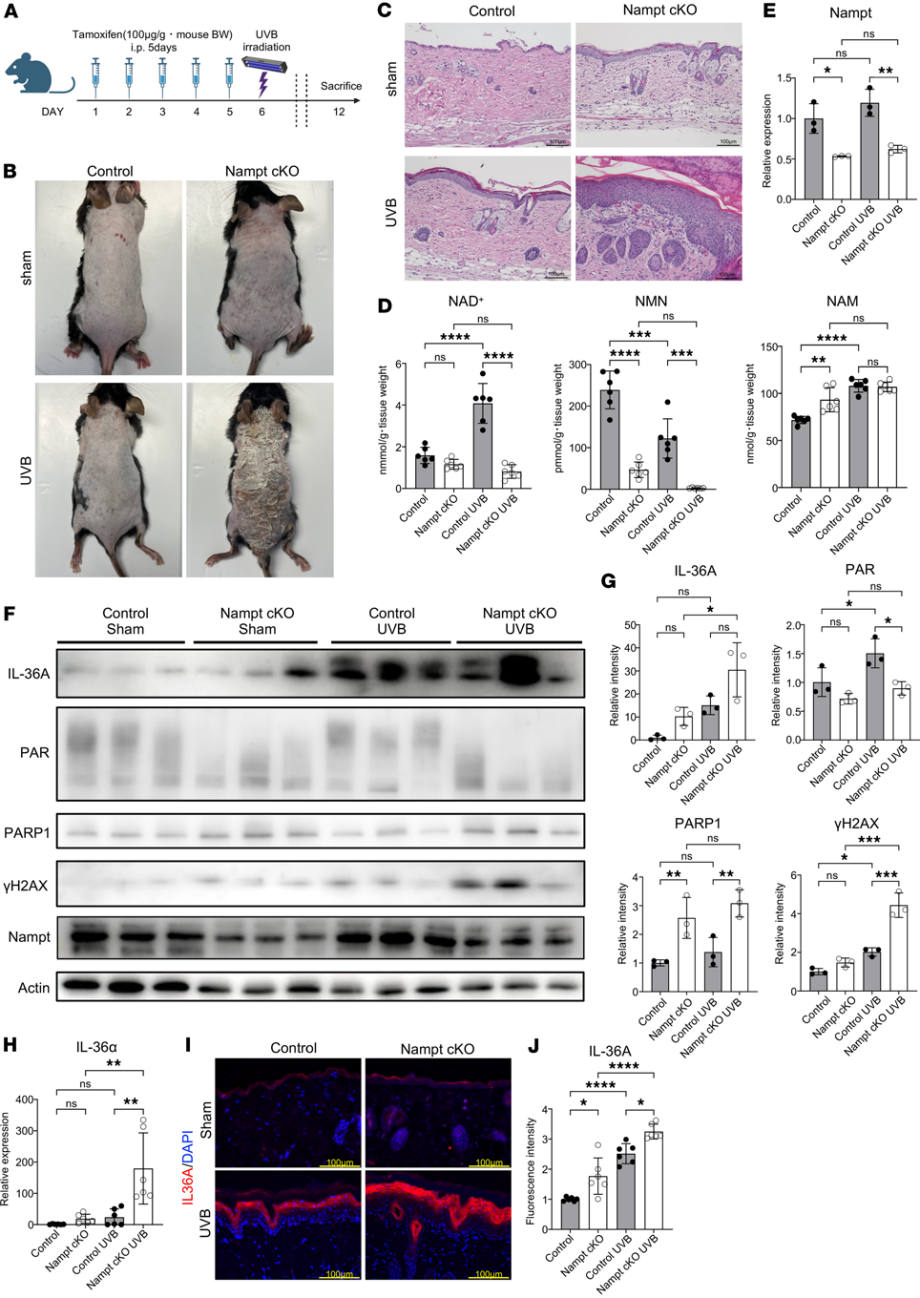
UVB-enhanced epidermal inflammation in Nampt-cKO mice. (**A**) Experimental time line showing tamoxifen administration, UVB irradiation, and sample collection for control and Nampt-cKO mice. (**B**) Representative clinical photographs of control and Nampt-cKO mice after UVB irradiation or sham treatment on Day 12. (**C**) Representative H&E-stained sections of control and Nampt-cKO skin after UVB irradiation or sham treatment on Day 12. Scale bars: 100 μm. (**D**) Levels of NAD^+^, NMN, and NAM in the epidermis of control and Nampt-cKO mice after UVB irradiation or sham treatment on Day 12, as measured by LC/MS. Data are shown as mean ± SEM. Two-way ANOVA with Tukey’s post hoc test. *n* = 6. (**E**) Nampt mRNA levels in the epidermis of control and Nampt-cKO mice after UVB irradiation or sham treatment on Day 12, as determined by qPCR. Data are shown as mean ± SEM. Two-way ANOVA with Tukey’s post hoc test. *n* = 6. (**F**) Western blot analysis for IL-36A, PAR, PARP1, γH2AX, and NAMPT in the epidermis of control and Nampt-cKO mice after UVB irradiation or sham treatment on Day 12. Pan-actin was used as a loading control. (**G**) Semiquantitative densitometric analysis of Western blots normalized to pan-actin using ImageJ. Data are shown as mean ± SEM. Two-way ANOVA with Tukey’s post hoc test. *n* = 3. (**H**) IL-36α mRNA levels in the epidermis of control and Nampt-cKO mice after UVB irradiation or sham treatment on Day 12, as determined by qPCR. Data are shown as mean ± SEM. Two-way ANOVA with Tukey’s post hoc test. *n* = 6. (**I**) Representative immunofluorescence images of IL-36A in control and Nampt-cKO skin after UVB irradiation or sham treatment on Day 12. Scale bars: 100 μm. (**J**) Quantification of IL-36A in immunofluorescence images using ImageJ. Data are shown as mean ± SEM. Two-way ANOVA with Tukey’s post hoc test. *n* = 6. *P < 0.05, **P < 0.01, ***P < 0.001, ****P < 0.0001.

Dermatitis in patients with pellagra is marked by photosensitive inflammation, indicating that UV exposure accelerates NAD^+^ depletion and DNA damage accumulation. To investigate this, we performed a UVB irradiation experiment on the Nampt-cKO mice. On Day 6 after tamoxifen treatment, the mice’s dorsal skin was exposed to a single dose of UVB (100 mJ/cm^2^) (Figure 4A). Nampt-cKO mice showed no visible skin inflammation on Day 12 without UVB exposure (sham group). However, UVB exposure worsened skin inflammation in these mice on Day 12 (Figure 4, B and C). We also measured NAD^+^ levels in the UVB-irradiated skin. UVB exposure significantly increased NAD^+^ levels in the control mice but not in the Nampt-cKO mice on Day 12 (Figure 4D). However, UVB exposure did not change the Nampt gene expression in either control or Nampt-cKO mice on Day 12 (Figure 4E). This is likely because elevated DNA damage induces a compensatory increase in NAD^+^ biosynthesis through pathways other than the salvage pathway. Western blot analysis showed that impaired PAR synthesis, increased γH2AX levels, and elevated IL-36A expression were more pronounced in the Nampt-cKO mice compared with the sham group (Figures 4, F and G). Analysis with qPCR (Figure 4H) and IHC (Figure 4, I and J) also confirmed that IL-36A was more significantly upregulated in the UVB-irradiated Nampt-cKO mice. These findings suggest that UVB irradiation worsens the phenotype in Nampt-cKO mice, with IL-36A overexpression contributing to the effect, further linking DNA damage to IL-36A expression.

### Percutaneous administration of NMN ameliorates skin inflammation in Nampt-cKO mice.

**Figure 5:**
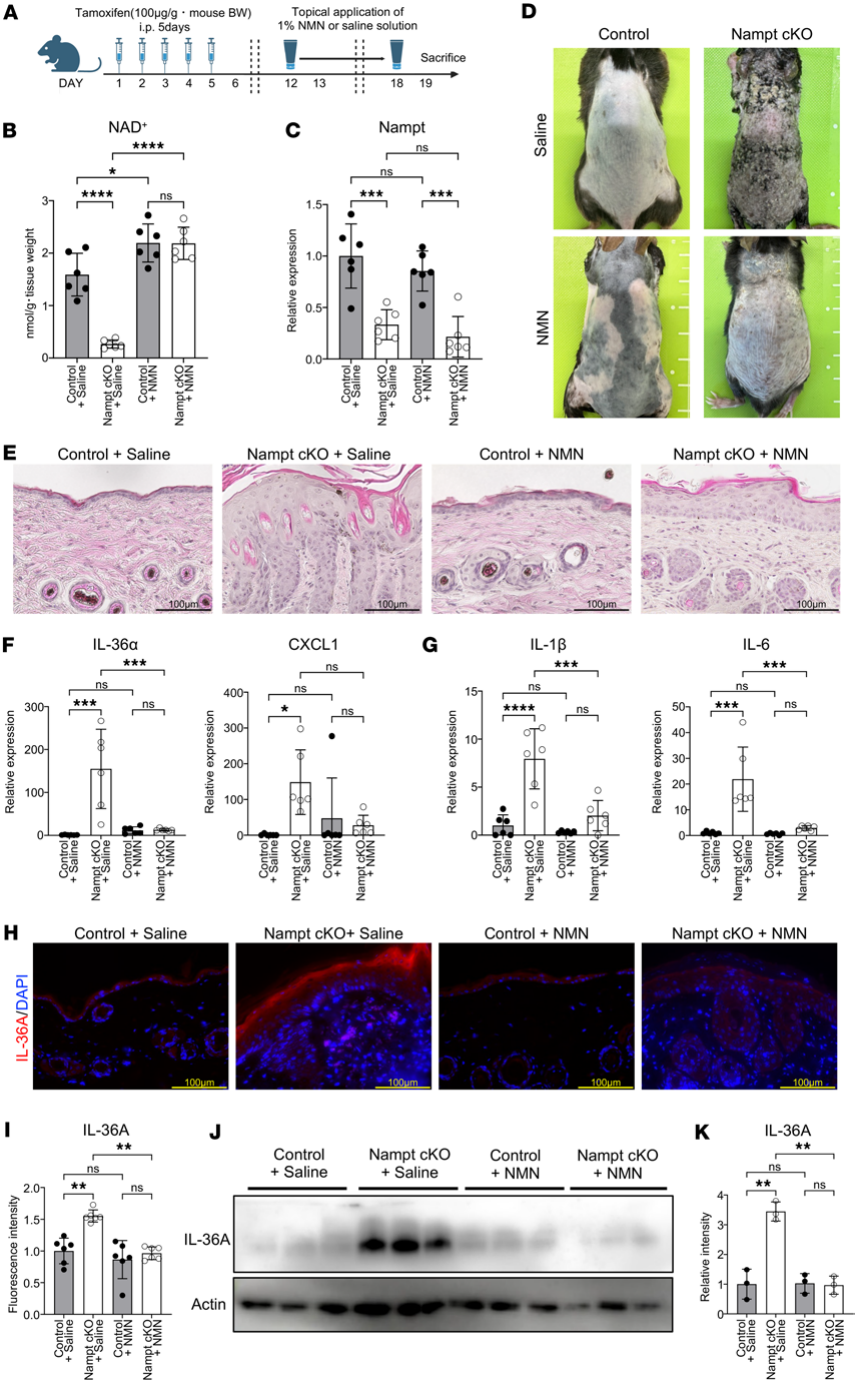
Topical NMN treatment ameliorates skin inflammation in Nampt-cKO mice. (**A**) Experimental time line showing tamoxifen administration, topical NMN treatment, and sample collection for control and Nampt-cKO mice. (**B**) NAD^+^ levels in the epidermis of control and Nampt-cKO mice with NMN or saline treatment on Day 19, as measured by LC/MS. Data are shown as mean ± SEM. Two-way ANOVA with Tukey’s post hoc test. *n* = 6. (**C**) Nampt mRNA levels in epidermis of control and Nampt-cKO mice with NMN or saline treatment on Day 19, as determined by qPCR. Data are shown as mean ± SEM. Two-way ANOVA with Tukey’s post hoc test. *n* = 6. (**D**) Representative clinical photographs of control and Nampt-cKO mice with NMN or saline treatment on Day 19. (**E**) Representative H&E-stained sections of control and Nampt-cKO mice with NMN or saline treatment on Day 19. Scale bars: 100 μm. (**F** and **G**) Gene expression levels of inflammation-related cytokines in epidermis of control and Nampt-cKO mice with NMN or saline treatment on Day 19, as determined by qPCR. Data are shown as mean ± SEM. Two-way ANOVA with Tukey’s post hoc test. *n* = 6. (**H**) Representative IHC images for IL-36A in the epidermis of control and Nampt-cKO mice with NMN or saline treatment on Day 19. Hematoxylin was used for counterstain. Scale bars: 100 μm. (**I**) Quantification of IL-36A in immunofluorescence images using ImageJ. Data are shown as mean ± SEM. Two-way ANOVA with Tukey’s post hoc test. *n* = 6. (**J**) Western blot analysis for IL-36A in the epidermis of control and Nampt-cKO mice with NMN or saline treatment on Day 19. Pan-actin was used as a loading control. (**K**) Semiquantitative densitometric analysis of IL-36A Western blots normalized to pan-actin using ImageJ. Data are shown as mean ± SEM. Two-way ANOVA with Tukey’s post hoc test. *n* = 3. *P < 0.05, **P < 0.01, ***P < 0.001, ****P < 0.0001.

Finally, we examined whether NAD^+^ replenishment could prevent skin inflammation in Nampt-cKO mice. We percutaneously administered NMN to Nampt-cKO mice from Day 12 to Day 18 after tamoxifen treatment (Figure 5A). Percutaneous administration of NMN significantly restored NAD^+^ levels in Nampt-cKO mice without altering Nampt gene expression (Figure 5, B and C). Saline-treated Nampt-cKO mice developed apparent skin inflammation by Day 18, whereas NMN-treated Nampt-cKO mice exhibited improved skin condition as early as Day 12 (Figure 5D). Histological analysis revealed markedly reduced epidermal hyperplasia in NMN-treated compared with saline-treated Nampt-cKO mice (Figure 5E). The expression of IL-36A and CXCL1 was significantly downregulated in NMN-treated Nampt-cKO mice (Figure 5F). Furthermore, the expression of inflammatory cytokines, including IL-1β and IL-6, was also significantly suppressed (Figure 5G). Suppression of IL-36A at the protein level in NMN-treated Nampt-cKO mice was confirmed by immunofluorescence microscopy (Figure 5, H and I) and Western blot analysis (Figure 5, J and K). Collectively, these findings demonstrate that NAD^+^ deficiency is a direct cause of IL-36 activation and subsequent skin inflammation. Moreover, percutaneous administration of NMN may represent a potential therapeutic option for photosensitive dermatitis, including pellagra.

## Discussion

In this study, we showed that the deletion of epidermal Nampt leads to spontaneous skin inflammation. Specifically, reduced NAD^+^ levels resulted in decreased PARP activity and increased γH2AX, indicating genomic stress accumulation. This DNA damage is believed to trigger IL-36 cytokine overproduction in keratinocytes, causing inflammation and hyperproliferation. In the Nampt-cKO mice, we also observed reduced COL17A1 expression and elevated TSLP levels. Previous studies have indicated that reduced COL17A1 disrupts epidermal stem cell function (26) and promotes TSLP-mediated inflammation (27). It is known that genomic stress–induced proteolysis leads to COL17A1 degradation (25). In this study, we found that IL-1β expression is induced in Nampt-cKO mice, and previous reports have shown that IL-1β promotes the degradation and shedding of COL17A1 (28, 29). These findings suggest that IL-1β induction resulting from impaired DNA damage repair in the skin of Nampt-cKO mice contributes not only to the upregulation of IL-36A but also to the induction of TSLP through COL17A1 degradation.

IL-36 is a key inflammatory mediator in GPP pathophysiology. It has been reported that IL-36 triggers inflammation via Th17 pathways in patients with GPP (18, 19), but the Nampt-cKO mice did not show signs of Th17 cell differentiation. This indicates that the inflammatory mechanism in Nampt-cKO mice differs from that in skin conditions like psoriasis and atopic dermatitis. Our data also show that the inflammation in Nampt-cKO mice was driven by innate immune cells, such as neutrophils and macrophages. While GPP also activates the IL-36 signaling pathway (30), its inflammation originates from autoimmune abnormalities. In contrast, inflammation in Nampt-cKO mice stems from keratinocyte stress. UV-induced DNA damage has previously been reported to induce IL-1β expression in the skin (31, 32). In our study, we also observed the induction of IκBζ, which is expected to enhance IL-36A expression. These findings suggest that, unlike GPP, the inflammatory response in this model is triggered predominantly by innate immune cells such as neutrophils, without substantial involvement of T cells.

This study was confined to animal models, and further research is necessary to translate these findings to humans. The characteristic of dermatitis in patients with pellagra is photosensitivity. The dermatitis in Nampt-cKO mice also exhibited photosensitive dermatitis. This implies that dysfunction of NAD^+^ metabolism may have significant effects on photosensitivity and oxidative stress responses in the skin. It has also been reported that skin Nampt levels and NAD^+^ decrease with aging (6, 33), which may contribute to age-related skin diseases. In this context, investigating the potential involvement of NAD^+^ metabolic dysfunction in photosensitive disorders — such as pellagra and chronic actinic dermatitis, which exhibits photosensitivity symptoms predominantly in the elderly — is a subject for future research. Moreover, NAD^+^ supplementation therapy has been suggested to be effective in many age-related diseases (3, 7), indicating that maintaining NAD^+^ levels in the skin may contribute to the suppression of inflammatory responses and aging phenomena. In this study, we demonstrated that percutaneous administration of NMN efficiently increased NAD^+^ levels in the absence of Nampt. Previous studies have reported that orally or intravenously administered NMN is readily degraded into NAM or nicotinic acid (34). Our current findings suggest that NMN can serve as a direct precursor for NAD^+^ synthesis in the skin and may represent a potential strategy to enhance NAD^+^ levels against photosensitizing dermatitis, including sunburn.

In summary, our study shows that NAD^+^ deficiency can trigger IL-36–mediated inflammation, offering insights into the role of NAD^+^ metabolism in skin inflammation. However, specific pathways connecting NAD^+^ deficiency, DNA damage, and IL-36 overproduction are still unclear and require further research. In addition, investigating the potential of NAD^+^ supplementation as a therapy could lead to new treatments for inflammatory skin diseases.

## Methods

### Sex as a biological variable.

Only male mice were used in this study; therefore, sex was not evaluated as a biological variable. It is unknown whether the findings are relevant for female mice.

### Generation of epidermis-specific Nampt-cKO mice.

Nampt floxed mice (C57BL/6J-Nampt^fl/fl^) (35) were crossed with KRT5-CreERT2 mice (C57BL/6J-Krt5^CreERT2^) (36) to generate epidermis-specific Nampt-cKO mice (Nampt^fl/fl^ KRT5-^CreERT2+^). Genotyping was performed by PCR to confirm the presence of the floxed Nampt allele and CreERT2 transgene. Primer sequences are provided in Supplemental Table 1 (supplemental material available online with this article; https://doi.org/10.1172/jci.insight.189177DS1).

### Tamoxifen administration.

To induce Cre-mediated recombination, 8-week-old male Nampt-cKO mice received i.p. injections of tamoxifen (Sigma-Aldrich, T5648) dissolved in corn oil (Sigma-Aldrich, C8267) at 20 mg/mL. Tamoxifen was administered at 100 μg/g body weight once daily for 5 consecutive days.

### UVB irradiation experiment.

Nampt-cKO mice and control mice received tamoxifen to induce recombination as previously described. On Day 6 after tamoxifen administration, mice were sedated with a general triple combination anesthetic (medetomidine, midazolam, and butorphanol) to minimize movement during the procedure. Following sedation, the dorsal skin was carefully shaved to ensure uniform exposure. A single dose of UVB (100 mJ/cm^2^) was irradiated to the shaved dorsal skin using a UVB lamp (280–320 nm). To localize the exposure, nontargeted areas were shielded during irradiation.

### Topical NMN treatment.

Nampt-cKO and control mice were sedated and dorsally shaved, as described above. NMN (Tokyo Chemical Industry, N1123) was dissolved in sterile 0.9 % saline (Otsuka Pharmaceutical Factory, 1326) to prepare a fresh 1 % (w/v) solution each day. From Day 12 to Day 18 after tamoxifen administration, 2 mL of this solution — corresponding to approximately 20 mg NMN per mouse — was applied once daily to the shaved dorsal skin with a fine-mist sprayer; vehicle controls received the same volume of saline. Four groups were evaluated (Control + Saline, Control + NMN, Nampt-cKO + Saline, Nampt-cKO + NMN; *n* = 6 per group). Dorsal skin was reshaved at necropsy, and tissues were collected on Day 19.

### Tissue preparation and H&E staining.

Skin samples were collected from the dorsal region of mice at specified time points (Day 0, 12, 19, and 26 after tamoxifen administration), fixed in 10% neutral-buffered formalin, processed, and embedded in paraffin. Sections (4 μm) were stained with H&E for histological evaluation.

### IHC and immunofluorescence (IF).

Paraffin-embedded skin sections were deparaffinized, rehydrated, and underwent antigen retrieval. Sections were blocked and incubated overnight at 4°C with primary antibodies for TSLP (Abcam, ab188766, polyclonal, 1:400), COL17A1 (Abcam, ab184996, clone EPR18614, 1:400), CD3 (Proteintsch, 17616-1-AP, polyclonal, 1:400), Ly6G (BD Biosciences, 550291, clone RB6-8C5, 1:200), F4/80 (Proteintsch, 28463-1-AP, polyclonal, 1:400), IL-36A (Abcam, ab269274, clone EPR23152-241, 1:200), and Ki67 (Abcam, ab16667, clone SP6, 1:400). For IHC, sections were treated with HRP-conjugated secondary antibodies (Abcam, ab214880, Polyclonal, 1:400) and developed with DAB substrate. For IF, Alexa Fluor–conjugated secondary antibodies (Invitrogen, A11034 and A10040, Polyclonal, 1:500) were used, followed by counterstaining with DAPI.

### Western blotting analysis.

Protein extracts from skin tissues were prepared in RIPA buffer with protease inhibitors. Equal amounts of protein were separated by SDS-PAGE, transferred to PVDF membranes, and probed with primary antibodies against NAMPT Bethyl (Bethyl, A300-372A, Polyclonal, 1:5,000), PARP1 (Active Motif, 36010, Polyclonal, 1:2,000), PAR (biotechne, 4336-BPC, Polyclonal, 1:1,000), COL17A1 (Abcam, ab184996, clone EPR18614, 1:1,000), γH2AX (Abcam, ab2893, Polyclonal, 1:1,000), and pan-actin (Cell Signaling, 8456, clone D18C11, 1:1,000). HRP-conjugated secondary antibodies (Cell Signaling, 7074, Polyclonal, 1:1,000) were used, and signals were detected using chemiluminescence (Nacalai Tesque, 07880 and 02230).

### qPCR.

Total RNA was extracted from skin tissues using TRI Reagent (Sigma-Aldrich, T9424) and purified with the RNeasy Mini Kit (Qiagen, 74106) with reference to previous reports (37). RNA was reverse transcribed into cDNA using ReverTra Ace qPCR RT Master Mix (TOYOBO, FSQ-301). qPCR was performed using THUNDERBIRD SYBR qPCR Mix (TOYOBO, QPS-201) on an ABI StepOnePlus Real-Time PCR System. Relative gene expression was calculated using the ΔΔCt method with Actb as the housekeeping gene. Primer sequences are provided in Supplemental Table 1.

### RNA-seq.

RNA-seq was performed by Gene Nex on Nampt-cKO and control mice at Day 19. Libraries were prepared using poly-A selection and sequenced on an Illumina NovaSeq 6000. Differential gene expression analysis was conducted following standard protocols. The list of differentially expressed genes (DEGs) is shown in Supplemental Table 2.

### NAD^+^ and its related metabolite quantification by LC/MS.

NAD metabolites in mouse tissues were quantified using LC/MS as previously reported (38). Frozen skin tissues were homogenized, metabolites were extracted, and samples were analyzed using an Agilent 6460 Triple Quadrupole LC/MS system. The MRM transitions and retention times for each metabolite are summarized in Supplemental Table 3.

### Statistics.

Statistical analyses were performed using GraphPad Prism software (version 10.0.0). Data are presented as mean ± SEM. Statistical significance was assessed using 2-tailed Student’s *t* test or 2-way ANOVA, followed by Tukey’s post hoc multiple-comparison test when applicable. *P* < 0.05 was considered statistically significant.

### Study approval.

All animal experiments were approved by the Animal Experimentation Ethics Committee of the University of Toyama (A2022MED-19 and A2025MED-05) and conducted in accordance with institutional guidelines for animal welfare as well as the *Guide for the Care and Use of Laboratory Animals* (National Academies Press, 2011).

### Data availability.

All raw RNA-seq data generated in this study have been deposited in the NCBI Gene Expression Omnibus (GEO) under accession number GSE284223 (https://www.ncbi.nlm.nih.gov/geo). All other datasets supporting the findings of this study are available from the corresponding author upon reasonable request.

## Author contributions

Conceptualization was contributed by T Seki, YY, TM, and TN. Data curation was contributed by T Seki. Formal analysis was contributed by T Seki and JK. Funding acquisition was contributed by HU, KY, TM, T Shimizu, and TN. Investigation was contributed by T Seki, YY, HU, KY, and MK. Methodology was contributed by YY, HU, KY, and MK. Project administration was contributed by T Seki, and TN. Resources were contributed by KY, T Shimizu, and TN. Visualization was contributed by T Seki, JK, and TN. Writing of the original draft was contributed by T Seki. Review and editing were contributed by T Seki and TN.

## Funding support

- KAKENHI from the Japan Society for the Promotion of Science (22H02630, 22K19289, 23K24762, and 25H01095.)
- Kobayashi Foundation.
- Takeda Science Foundation.
- Tamura Science & Technology Foundation.
- Takeda Medical Research Foundation.
- Takahashi Industrial and Economic Research Foundation.
- Japan Foundation for Applied Enzymology.
- AMED-PRIME (grants no. 24gm6710007h0003 and 25gm6710007h0004).
- AMED-CREST (grant no. JP21gm1410010).
- Japan Science and Technology Agency Precursory Research for Embryonic Science and Technology (PRESTO) (JPMJPR214C).

## Supplementary Material

Unedited blot and gel images

Supplemental table 1

Supplemental table 2

Supplemental table 3

Supporting data values

## Version 1 02/10/2026

In-Press Preview

## Version 2 03/23/2026

Electronic publication
