# Supplementary material for: Epidermal NAD+ deficiency induces IL-36–mediated skin inflammation and acanthosis
Source: JCI Insight. 2026 Feb 10;11(6):e189177. doi: 10.1172/jci.insight.189177 (PMC13043086; doi:10.1172/jci.insight.189177)

Fig1C

Nampt　Day12

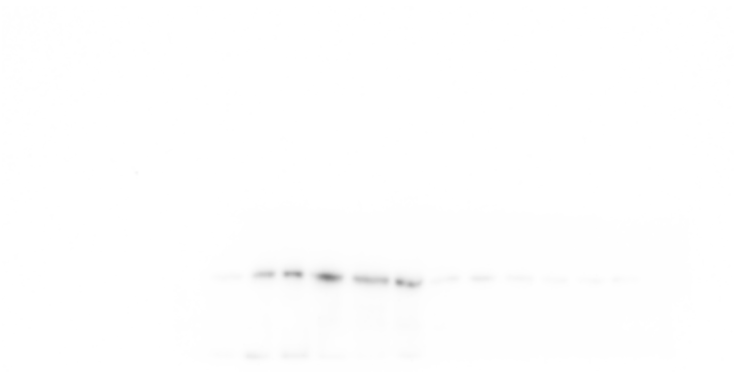

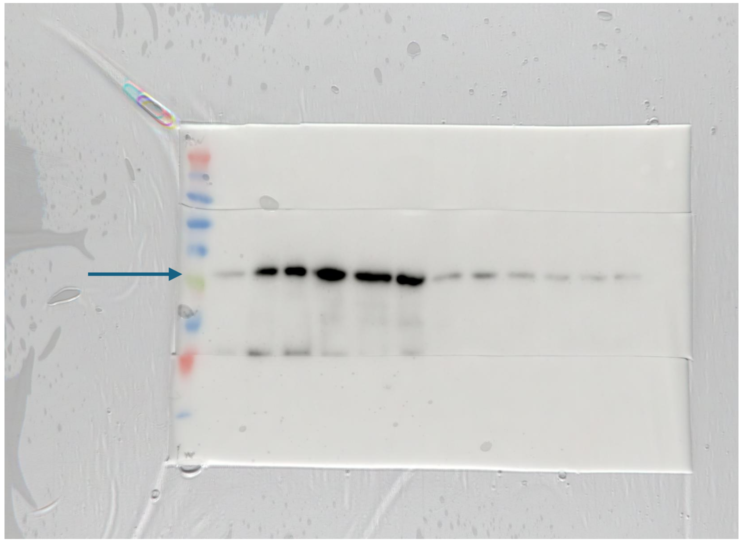

Fig1C

Actin Day12

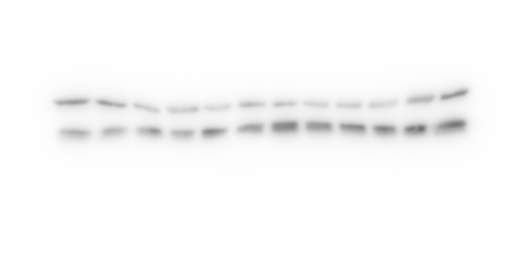

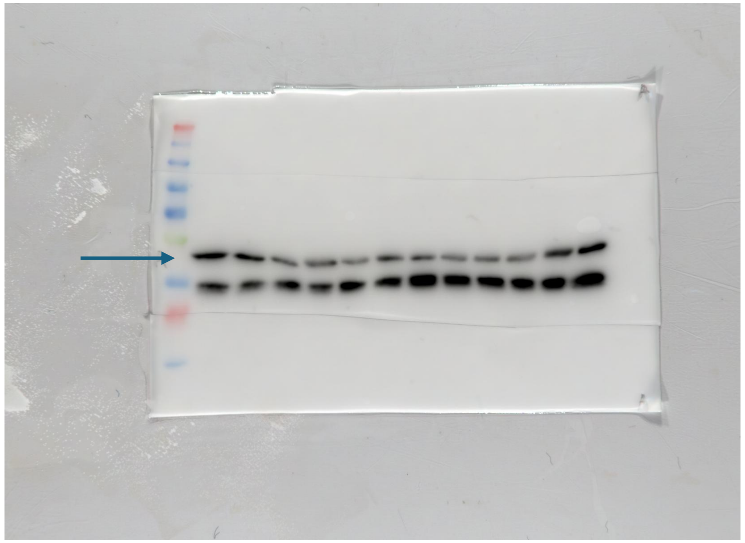

Fig1C

Nampt　Day19

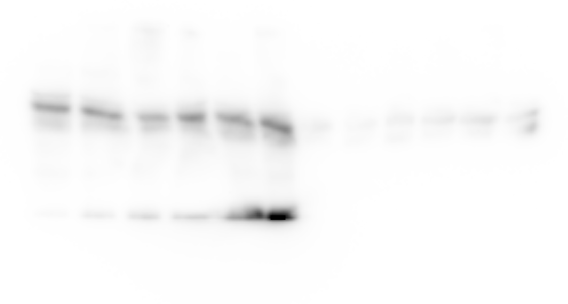

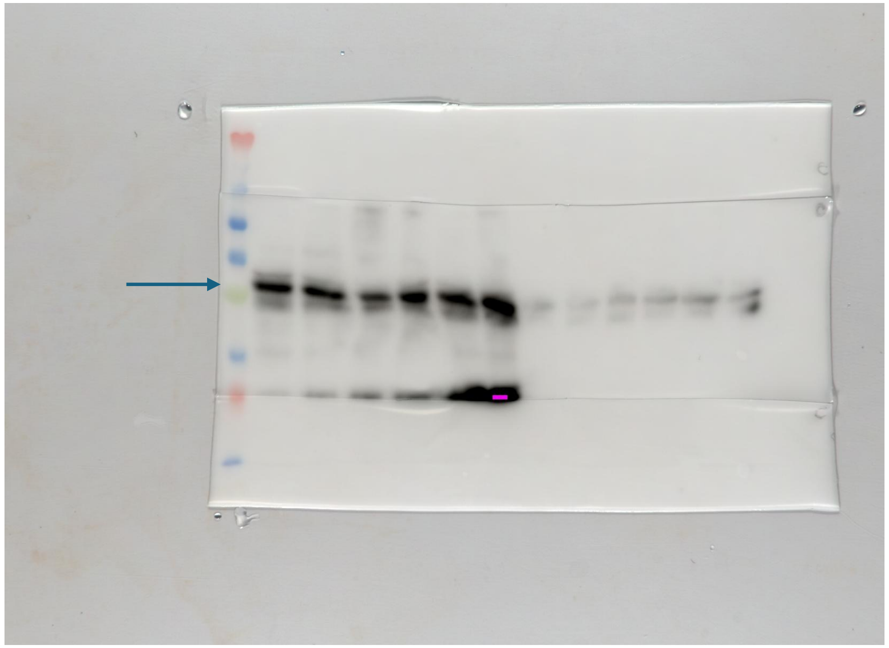

Fig1C

Actin Day19

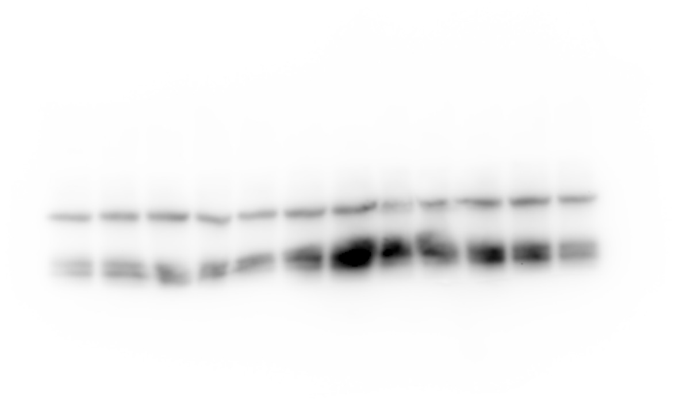

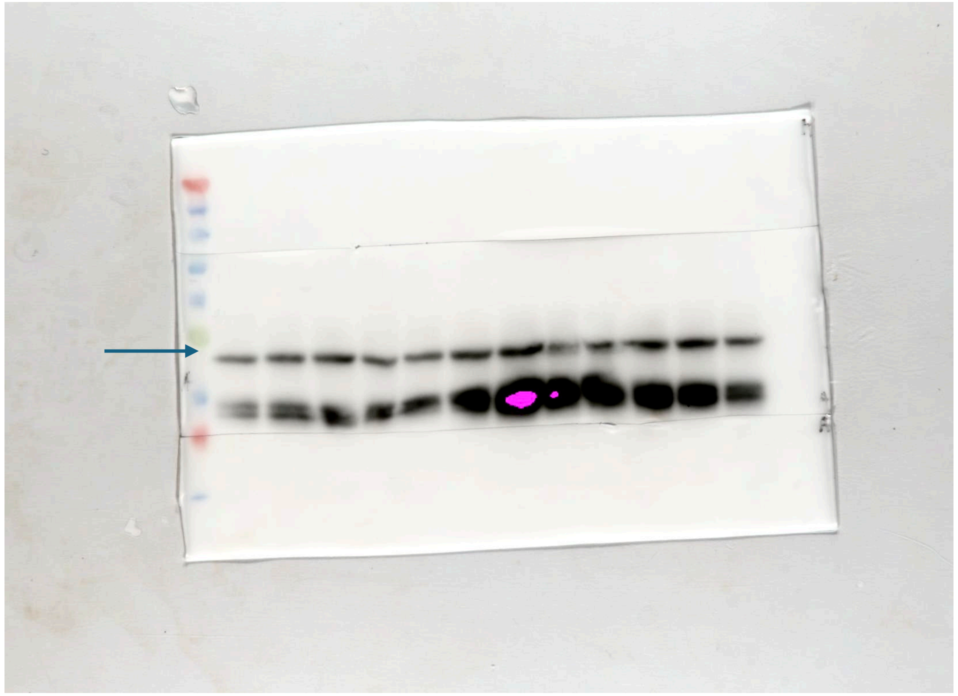

Fig1C

Nampt　 Day26

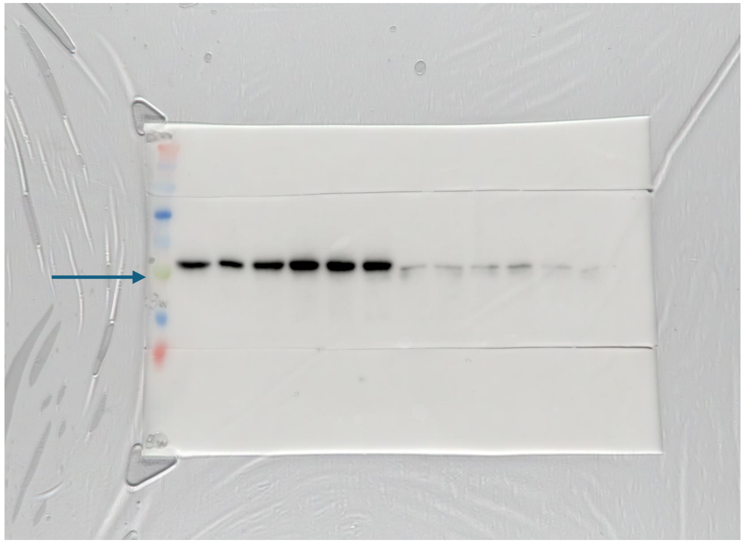

Fig1C

Actin　　Day26

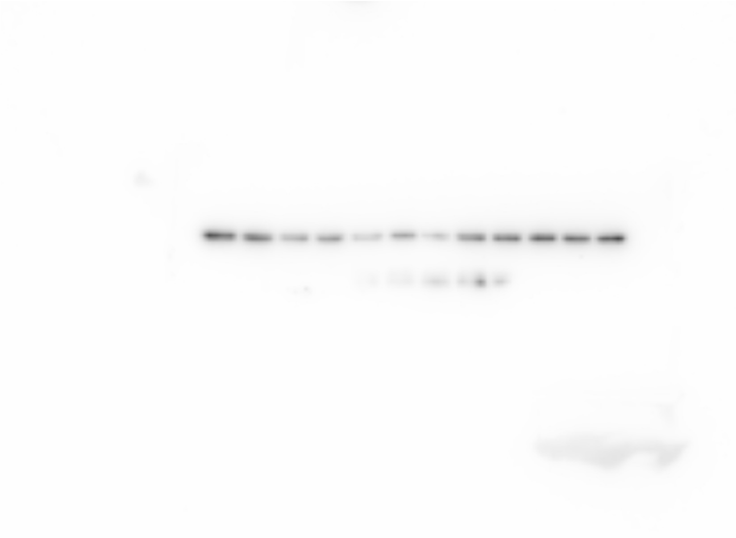

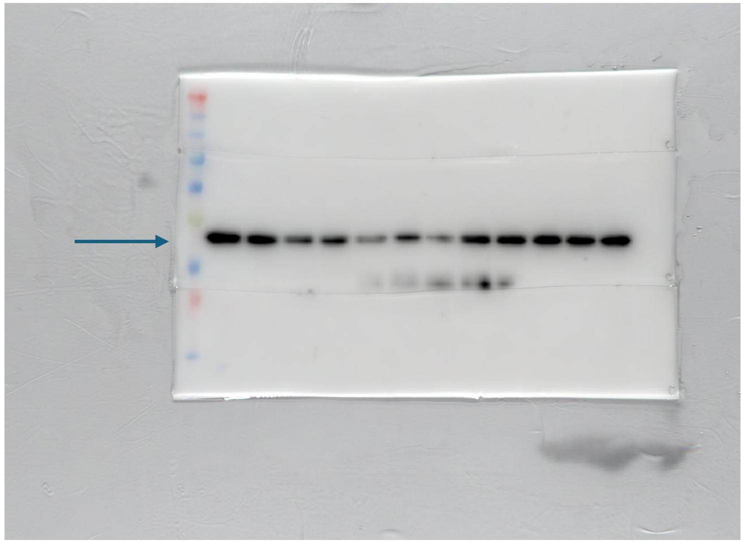

Fig3A

PAR

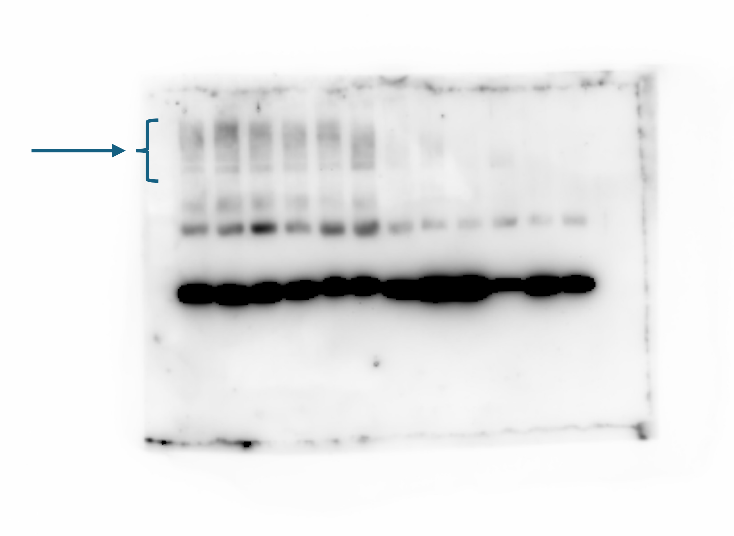

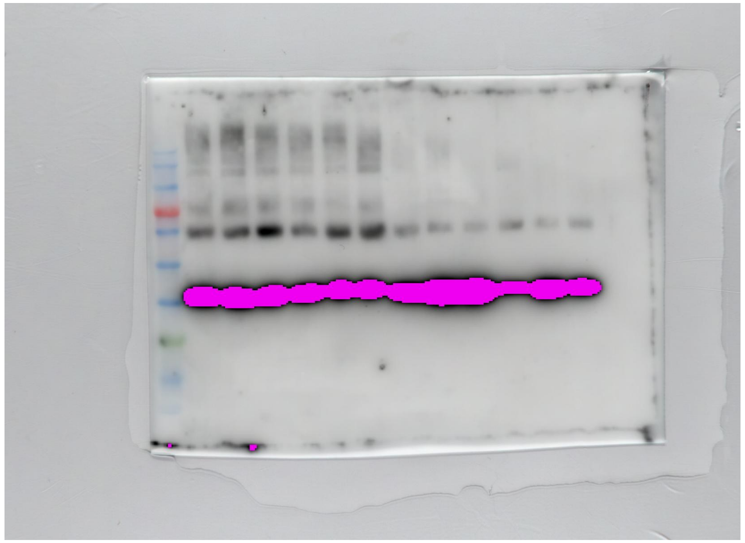

Fig3A

PARP1

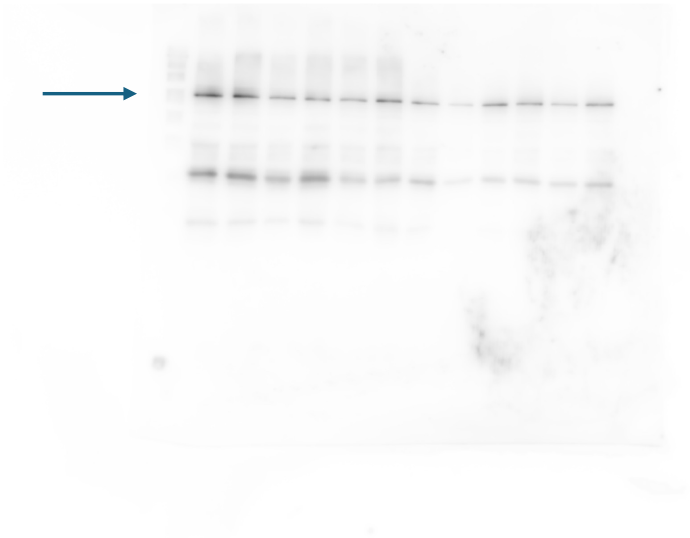

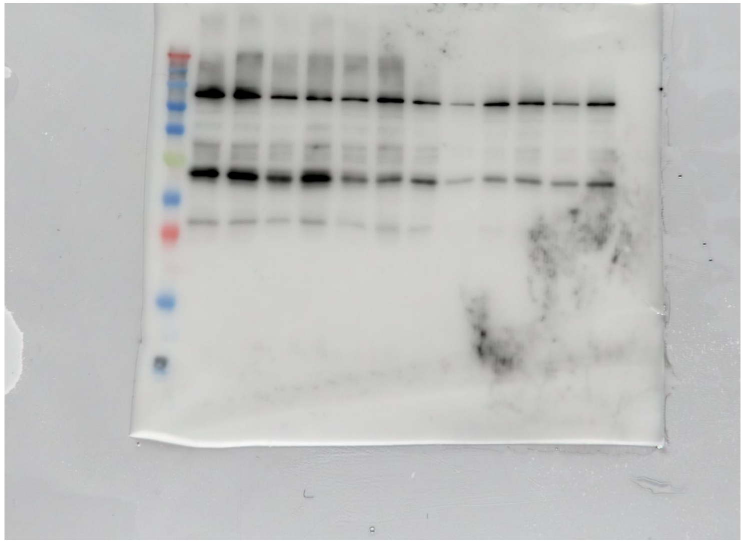

Fig3A

γ H2AX

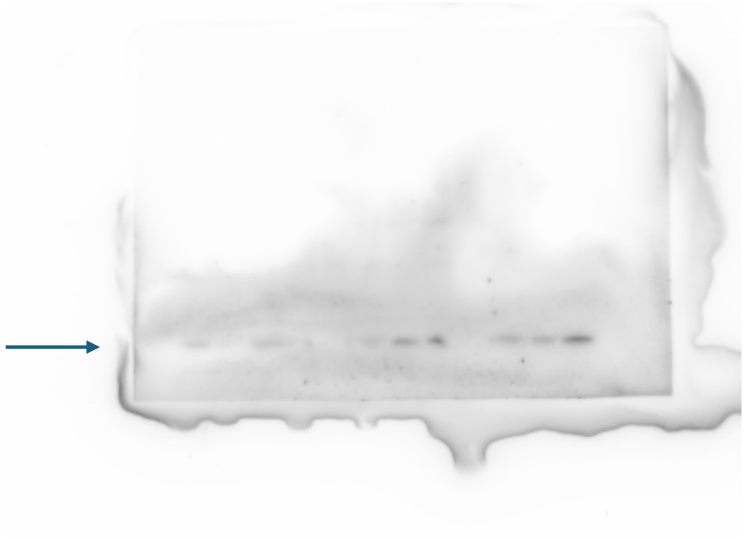

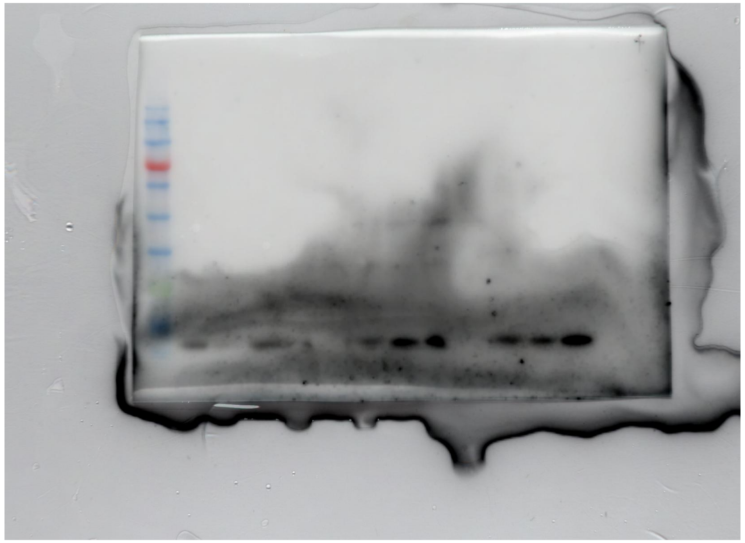

Fig3A

COL17A1

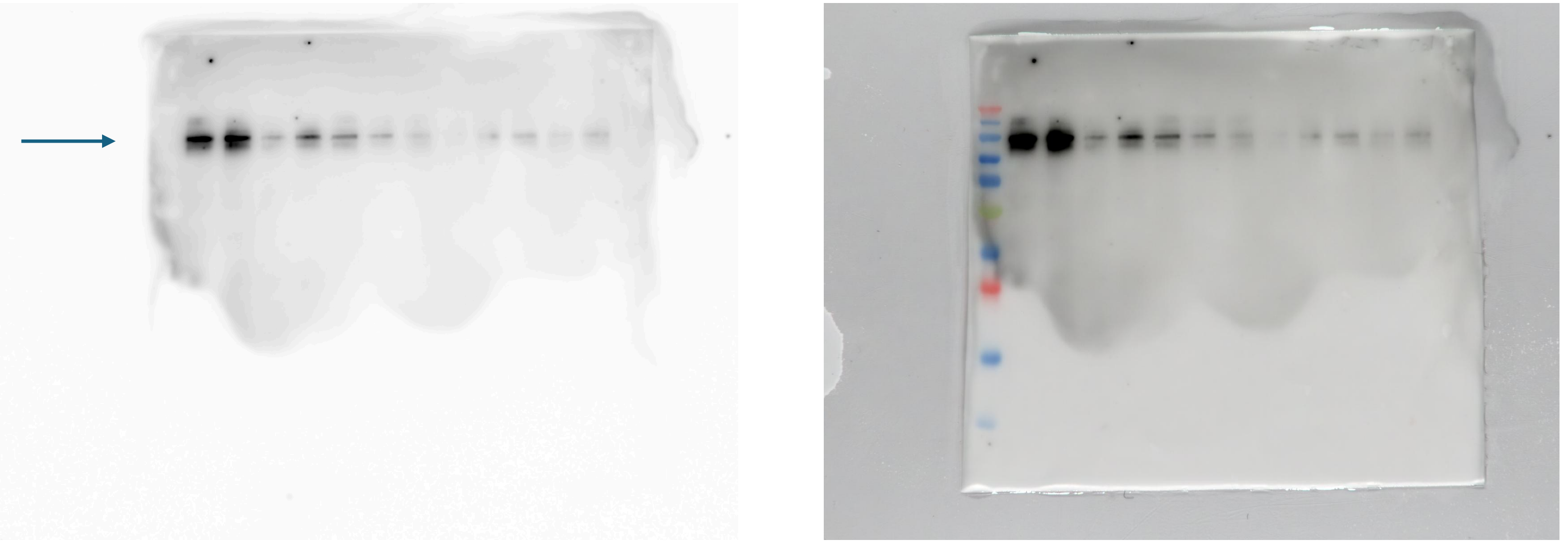

Fig3A

IL36A

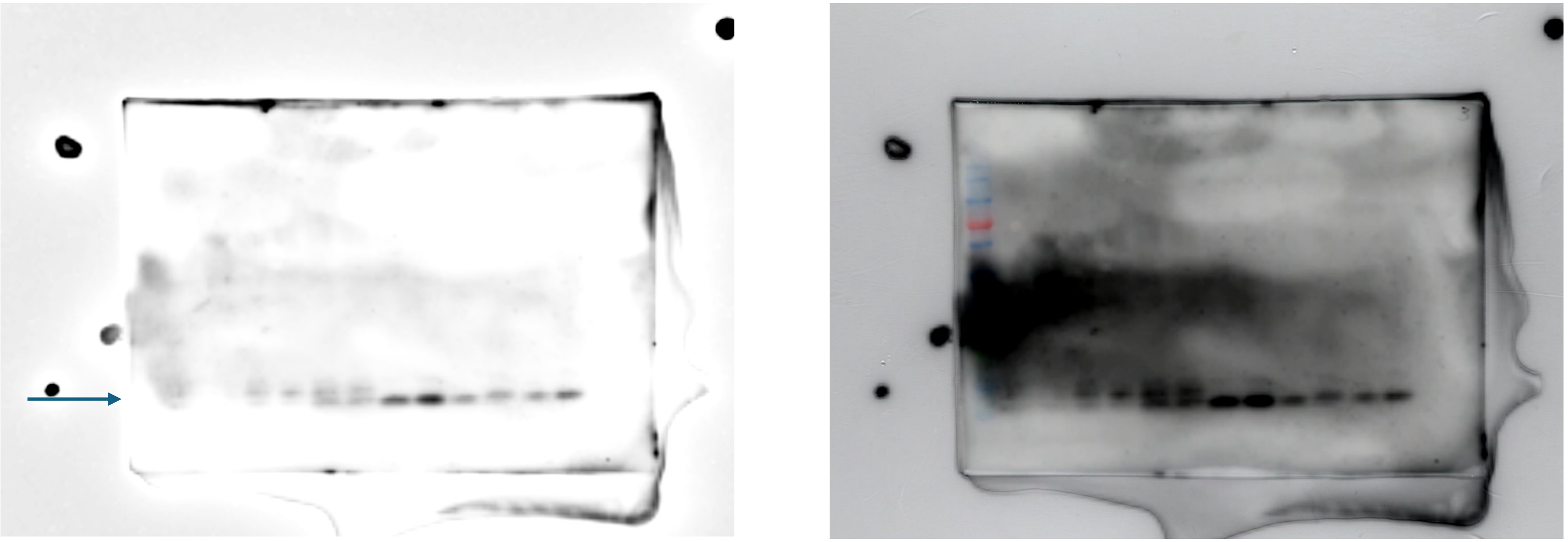

Fig3A

Pan Actin

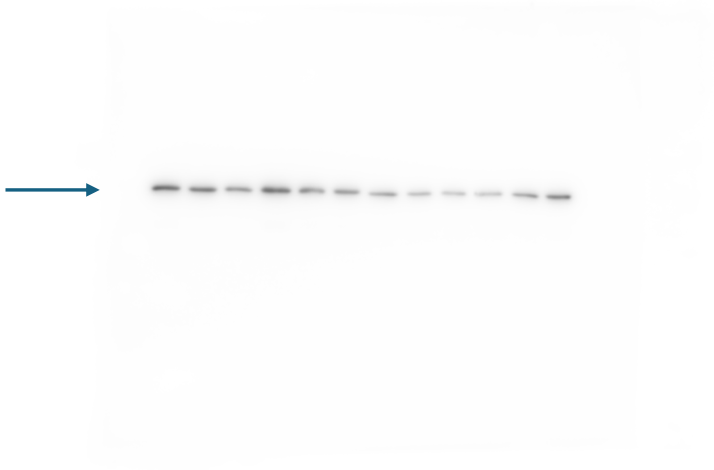

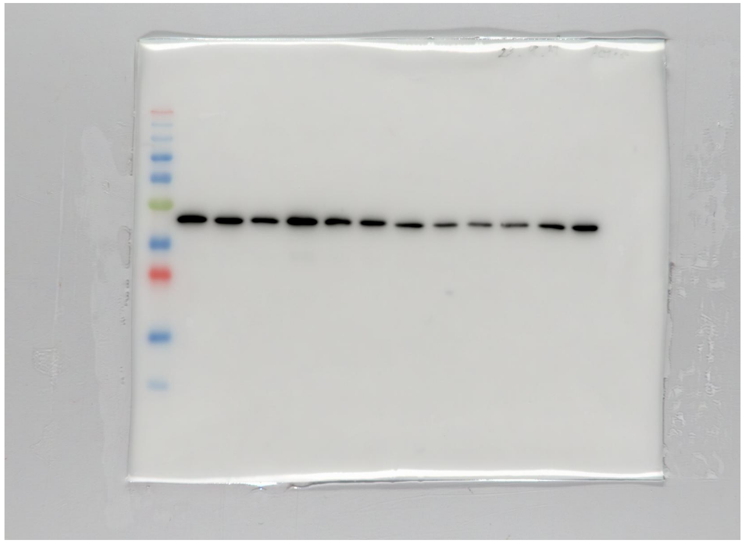

Fig4F

IL36A

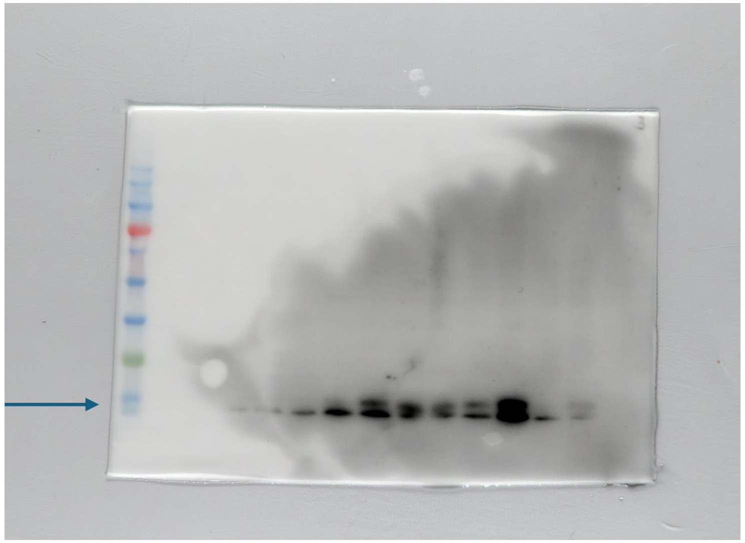

Fig4F

PAR

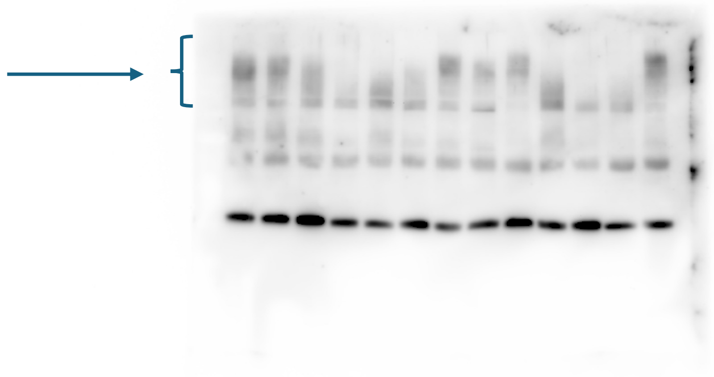

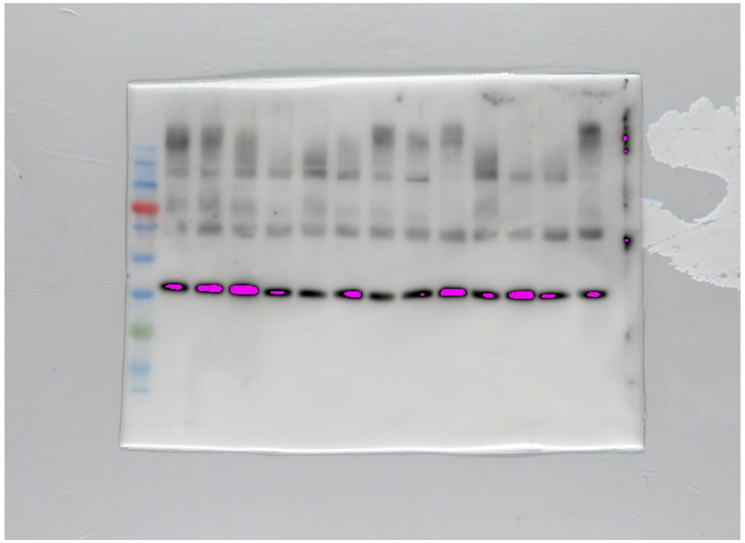

Fig4F

PARP1

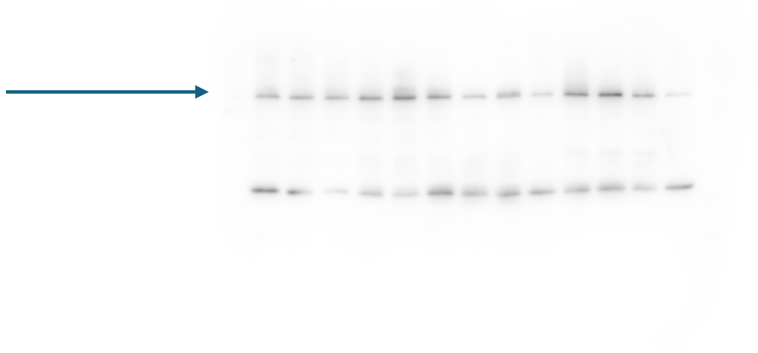

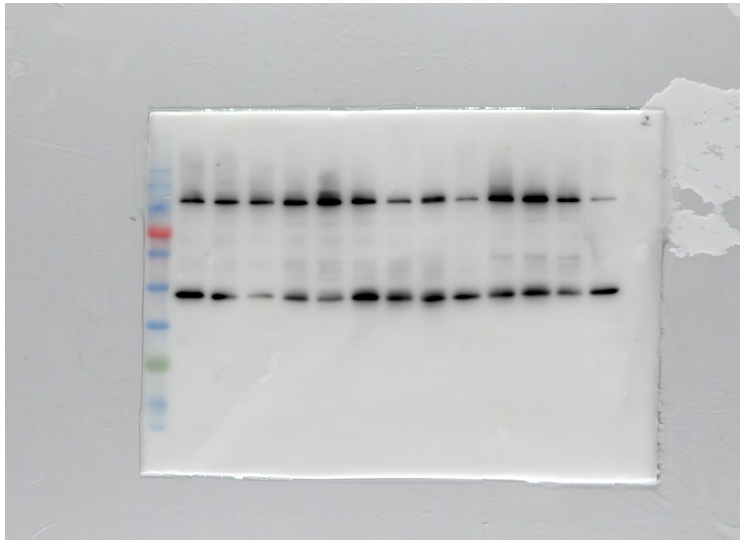

Fig4F

γ H2AX

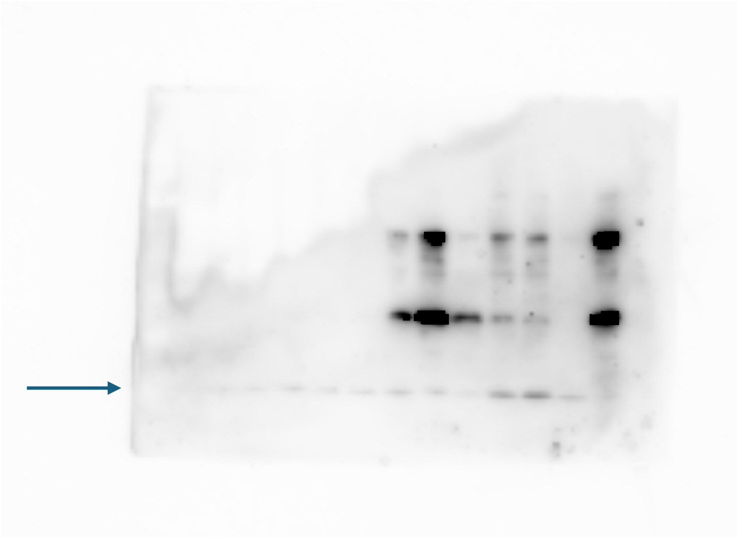

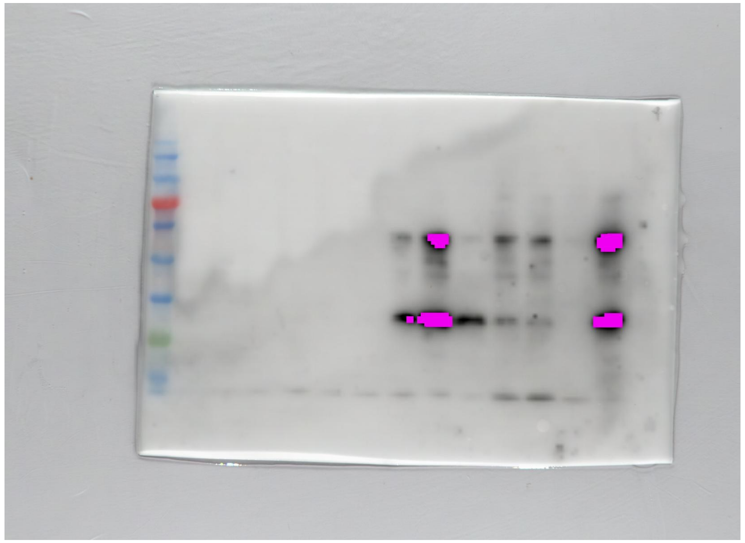

Fig4F

NAMPT

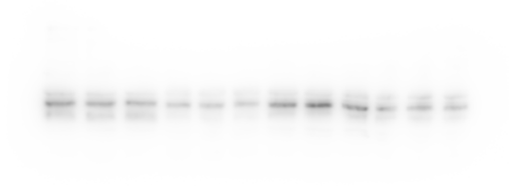

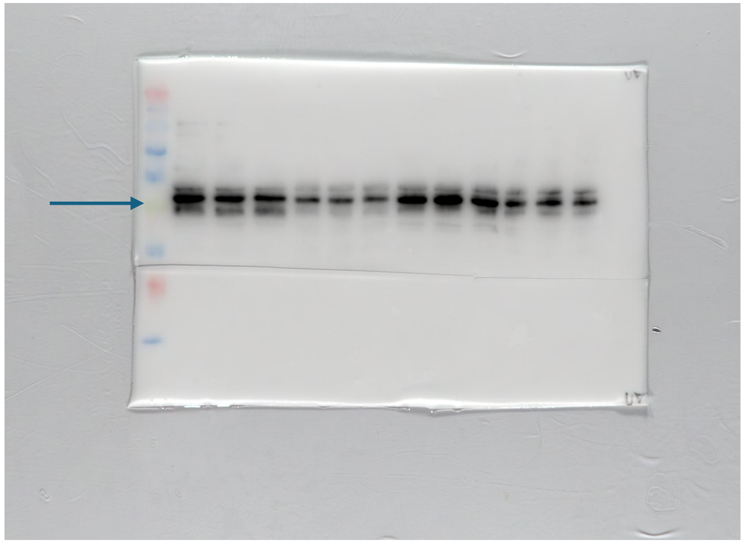

Fig4F

Pan Actin

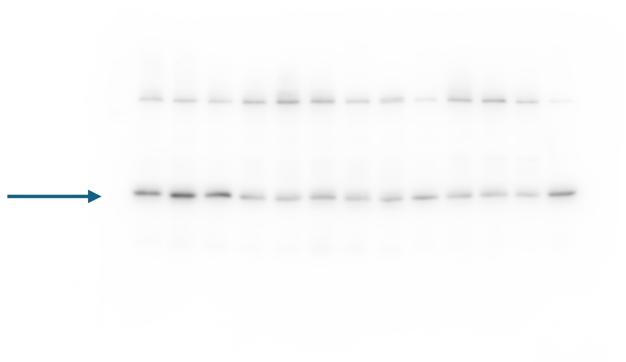

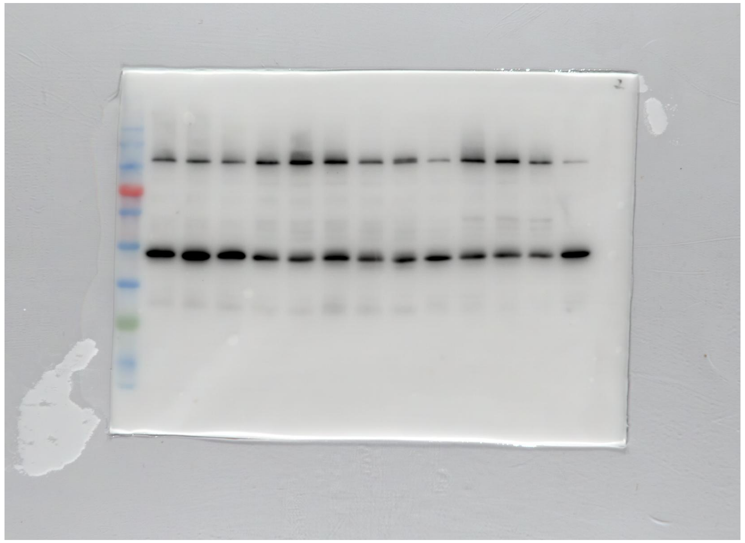

Fig5J

IL36A

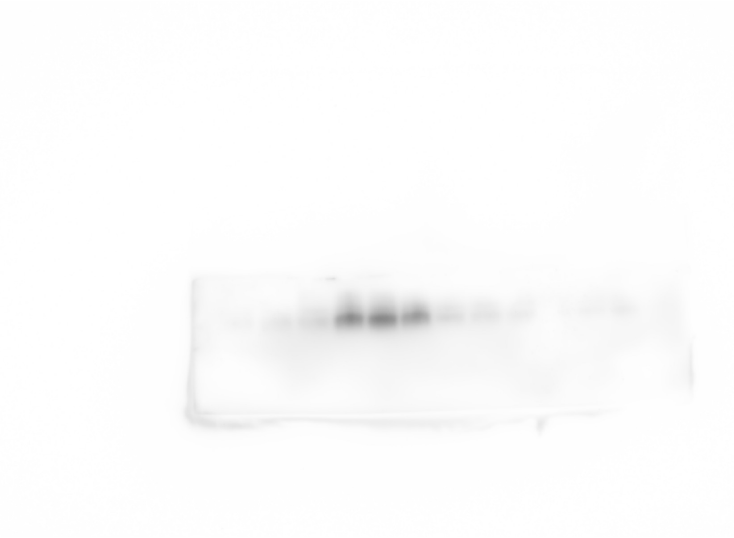

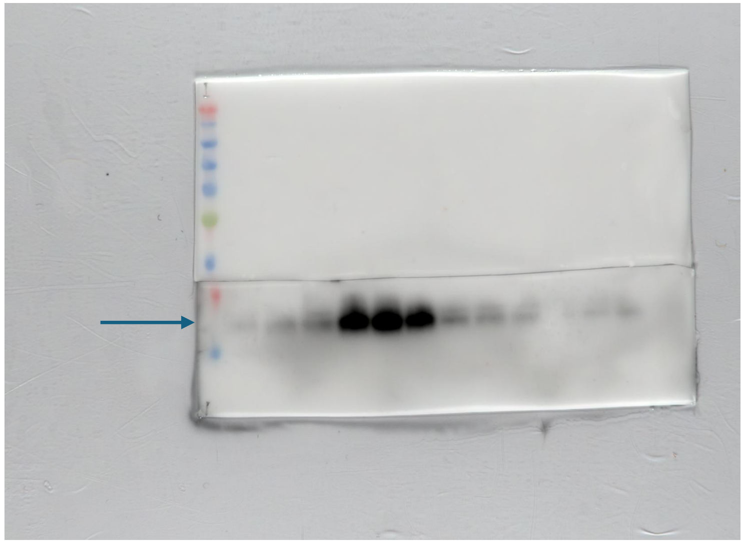

Fig5J

Actin

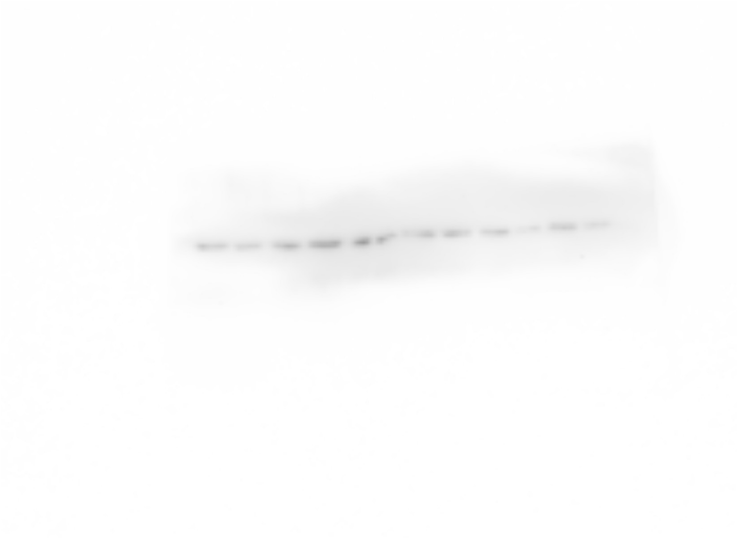

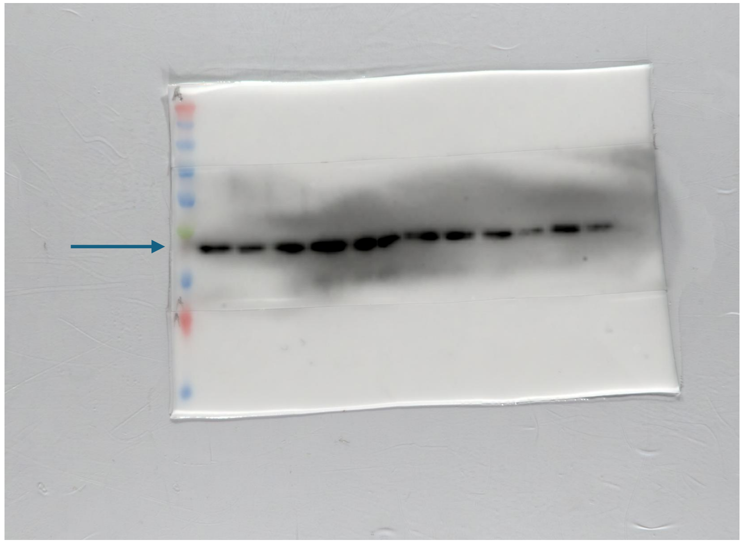

Supplement: Unedited blot and gel images [file jciinsight-11-189177-s033.pdf]
